# The phantastic organ: predictive processing in the brain

**DOI:** 10.3389/fpsyg.2026.1901293

**Published:** 2026-09-25

**Authors:** Karl Friston, Thomas Parr

**Affiliations:** 1 Queen Square Institute of Neurology, University College London, London, United Kingdom; 2 Nuffield Department of Clinical Neurosciences, University of Oxford, Oxford, United Kingdom

**Keywords:** active inference, active learning, Bayesian brain, predictive coding, predictive processing

## Abstract

Neuroscience has witnessed a curious inversion over the past decades, with a switch from views of the brain as abstracting information from sensory input to a view of the brain as a constructive organ, actively generating explanations for – and sampling – the sensorium. Perhaps this inversion is not strange, but a reversion to the ‘facts of perception’ as laid out by Helmholtz. His enduring view of perception as inference – to the best explanation – now dominates the neurosciences in the form of the Bayesian brain paradigm and the Free Energy Principle. This review follows the story from Helmholtz’s unconscious inference to modern day active inference. In particular, it uses the history of predictive coding – and how it has been generalized over the years – to furnish a framework that brings theory and empirical study in close contact, through generating mechanistic (anatomical and physiological) hypotheses about sense-making in the brain.

## 1 Introduction

*“…by the power of phantasy we see Colors in a Dream, or a mad Man sees things before him which are not there; or when we see Fire by striking the Eye, or see Colors like the Eye of a Peacock’s Feather, by pressing our Eyes in either corner whilst we look the other way.” – Isaac Newton, 1704, The First Book of Opticks Part II, p118*

Predictive coding can mean different things in different disciplines. It is therefore interesting to track the history of predictive coding from its inception to see the different ways it underwrites current thinking about functional brain architectures; ranging from synaptic self-organization to structure learning. The increasing compass of predictive coding presents great opportunities but also challenges. The opportunities at hand are the ability to unite disciplines and perspectives on common ground. The challenges rest on understanding one discipline’s view of predictive coding from the perspective of another. The aim of this review is to foreground the opportunities (and challenges) by unpacking the many aspects of predictive coding as they have emerged over time.

The core theme that ties all these aspects together is that the brain is a ‘phantastic’ organ, in the sense that its function depends upon an internal model of an external reality. In effect, this model generates an internal reality. The ‘ph’ spelling of phantasy emphasizes both an archaic English spelling used by Newton (see above) to describe forms of visual imagery not directly attributable to the properties of light, and links to (psychoanalytic) ideas from developmental psychology (Isaacs, 2018), in which an unconscious internal ‘psychic’ reality is adapted to an external reality. The imperative to align one’s internal model with their external world is central to each of the themes we examine in this paper.

In what follows, we begin with advances in theoretical physics and information theory in the mid-20th century, which portended the development of predictive coding – first as a statistical device for data compression and then as a theory of neural circuitry. Implicit in this theory is the notion that our brains engage in a kind of inference about the causes of our sensations. The mechanistic implications of this idea make sense of the laminar connectivity patterns observed within and between regions of cerebral cortex. This allows one to interpret ‘descending’ axonal projections from multimodal cortical to primary sensory regions of the brain as conveying predictions that are corrected by prediction errors communicated by the reciprocal ‘ascending’ projections.

We next turn to precision – an important quantity that helps to determine the confidence with which a prediction is made and therefore the relevance of different kinds of prediction error. Neurobiologically, precision manifests as a gain-like synaptic modulation of neurons representing prediction errors (i.e., superficial pyramidal cells), effectively turning up or down the volume of the prediction error signal conveyed to higher cortical regions. As our brains optimize precision, we see the emergence of phenomena like representational sharpening, sensory attenuation and selective attention. Interestingly, aberrant precision estimation has been implicated in a wide range of neurological and psychiatric conditions, underlining the translational potential of predictive coding.

At this point in the story, one is well-placed to account for a range of neuroanatomical and physiological findings, but there is a key ingredient missing: action. Here, one must engage with the ways in which our brains account for an itinerant world, including changes that we ourselves bring about. In doing so, we arrive at *active inference* in which one can resolve precision-weighted prediction errors not only through altering our predictions but also through action, so that sensory samples conform to descending predictions. Physiologically, this calls for circuitry resembling that of a spinal or brainstem reflex arc that ‘corrects’ proprioceptive input from stretch receptors by altering muscle tone. With these ingredients in place – prediction error minimization, precision modulation, and reflexive fulfillment of predictions through action – one can start to understand the links between predictive processing, reward learning, and even the recent re-emergence of predictive coding in machine learning.

Ultimately, the above turn out to follow from the same variational principles developed in the 1950s. With this understanding, one can revisit this history in reverse to understand how each of these developments is in fact a special case of the Free Energy Principle. One can then go further and use this principle to point the way forward in understanding how we select among alternative futures (i.e., how we plan based upon the anticipated consequences of our actions) and even how we optimize the structure of the internal world model under which we predict, perceive, and act upon our environment.

## 2 Predictive coding: a brief history

Two papers were published in 1955 that were to set the scene for a formal understanding of predictive processing in the brain some half-century later. The first introduced a simple and powerful path-integral approach to inference (Feynman, 1955) that would be later known as the Feynman variational principle, variously referred to as the Jensen-Feynman or Gibbs-Bogoliubov-Feynman inequality (Wouters and Saeys, 2003). The second heralded the inception of predictive coding (Elias, 1955a), following key developments in information theory (Bode and Shannon, 1950). Decades later, it transpired that both papers offered complementary answers to the same question: how do we make sense of data?

Predictive coding was originally developed as a powerful compression technique, that is still used in speech analysis today (Atal, 2006). The notion of compression speaks to an intimate relationship between (Bayes) optimal communication, information theory and algorithmic complexity. Examples include the compression implicit in minimum description and message length formulations of efficient communication and representation (Hinton and Zemel, 1993; Wallace and Dowe, 1999). Indeed, this line of thinking led to Solomonoff induction and notions of universal – albeit incomputable – computation (Hutter, 2006; Solomonoff, 2009). A more pragmatic line of thinking can be traced through the principle of maximum efficiency that rests upon maximizing mutual information, under constraints (Linsker, 1990; Bialek et al., 2001; Simoncelli and Olshausen, 2001; Ay et al., 2008; Ruffini, 2017). For some, the implicit minimisation of complexity has played a foundational role in approaches to artificial intelligence and machine learning (Schmidhuber, 2010; Ha and Schmidhuber, 2018). From a neuroscience perspective, these notions emerged as the principle of minimum redundancy, when considering the functional architecture of the visual cortex (Barlow, 1961; Barlow, 1974), with attendant notions of inference to the best explanation, in terms of perception as inference or hypothesis testing (Gregory, 1968, 1980). See Figure 1.

**FIGURE 1:**
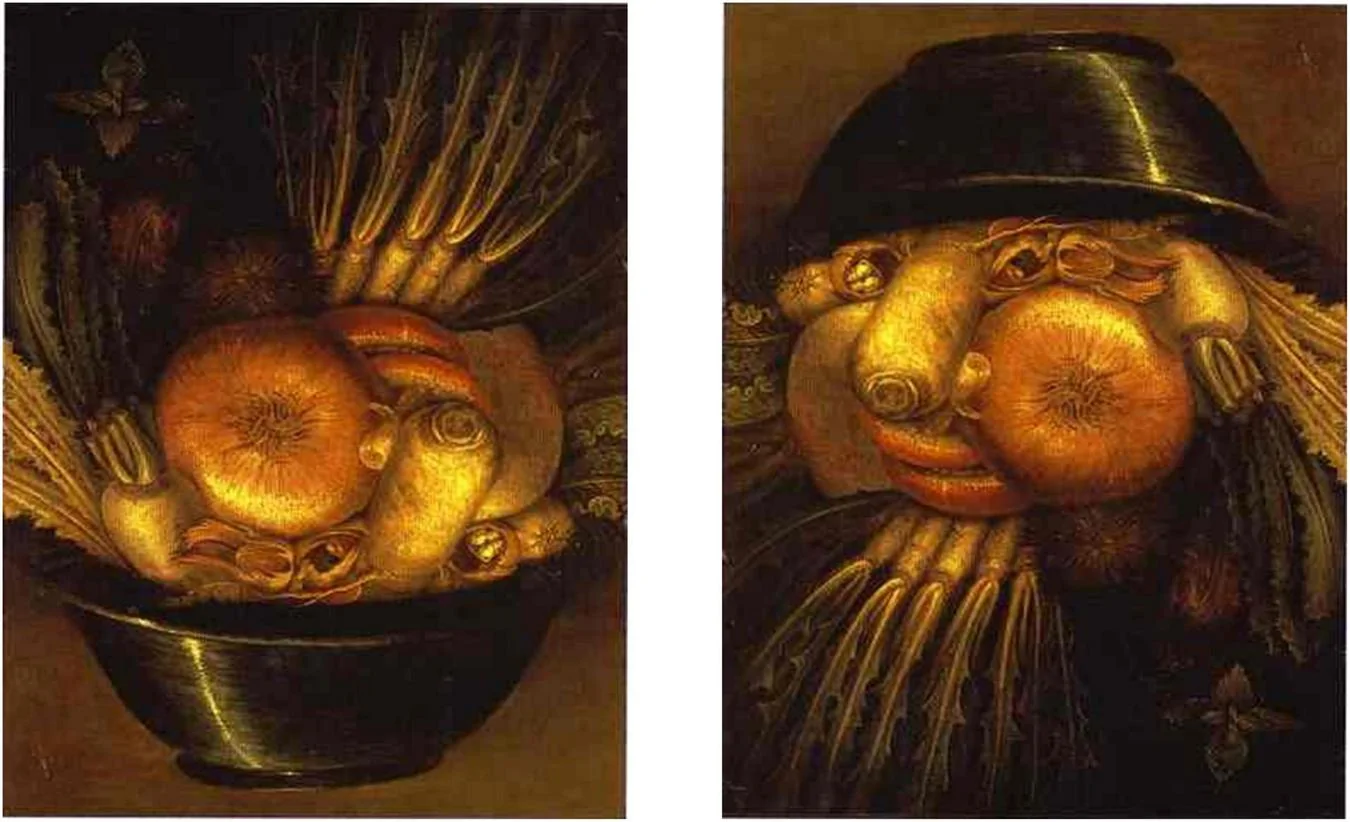
*Giuseppe Arcimboldo*, The Vegetable Gardener (c.1590). Oil on panel. Our percepts are constrained by what we expect to see and the hypotheses that can be called upon to explain our sensory input. Arcimboldo, a 16th century Milanese artist – a favorite of the Viennese – illustrates this dramatically by using fruits and vegetables to create faces in his paintings (Kandel, 2012). The point here is that if you see a bowl of fruit on the left but a face on the right, you brought this hypothesis, inference, explanation, illusion or phantasy (sic) to the table, to explain your sensory impressions.

But what about predictive coding *per se*? The principles of predictive coding were first applied in neurobiology to neuronal circuits in the retina in the 1980s (Srinivasan et al., 1982) and then generalized to hierarchical visual processing in the 1990s. The most explicit early formulation was provided by David Mumford (Mumford, 1992), who pre-empted subsequent formulations with remarkable precision:

“*I put forward a hypothesis on the role of the reciprocal, topographic pathways between two cortical areas, one often a ‘higher’ area dealing with more abstract information about the world, the other ‘lower’, dealing with more concrete data. The higher area attempts to fit its abstractions to the data it receives from lower areas by sending back to them from its deep pyramidal cells a template reconstruction best fitting the lower level view [prediction]. The lower area attempts to reconcile the reconstruction of its view that it receives from higher areas with what it knows, sending back from its superficial pyramidal cells the features in its data which are not predicted by the higher area [prediction errors]. The whole calculation is done with all areas working simultaneously, but with order imposed by synchronous activity in the various top-down, bottom-up loops.*” [Our insertions] (Mumford, 1992)

All the key architectural themes would re-emerge in subsequent formulations (Rao and Ballard, 1999; Grossberg, 2007; Friston K. J., 2008; Spratling, 2008; Bastos et al., 2012); ranging from recurrent counter-streams of descending predictions and ascending prediction errors in cortical hierarchies to an emphasis on deep (i.e., the hierarchical) architectures with local computations at each level. See Figure 2 and Box 1.

**FIGURE 2:**
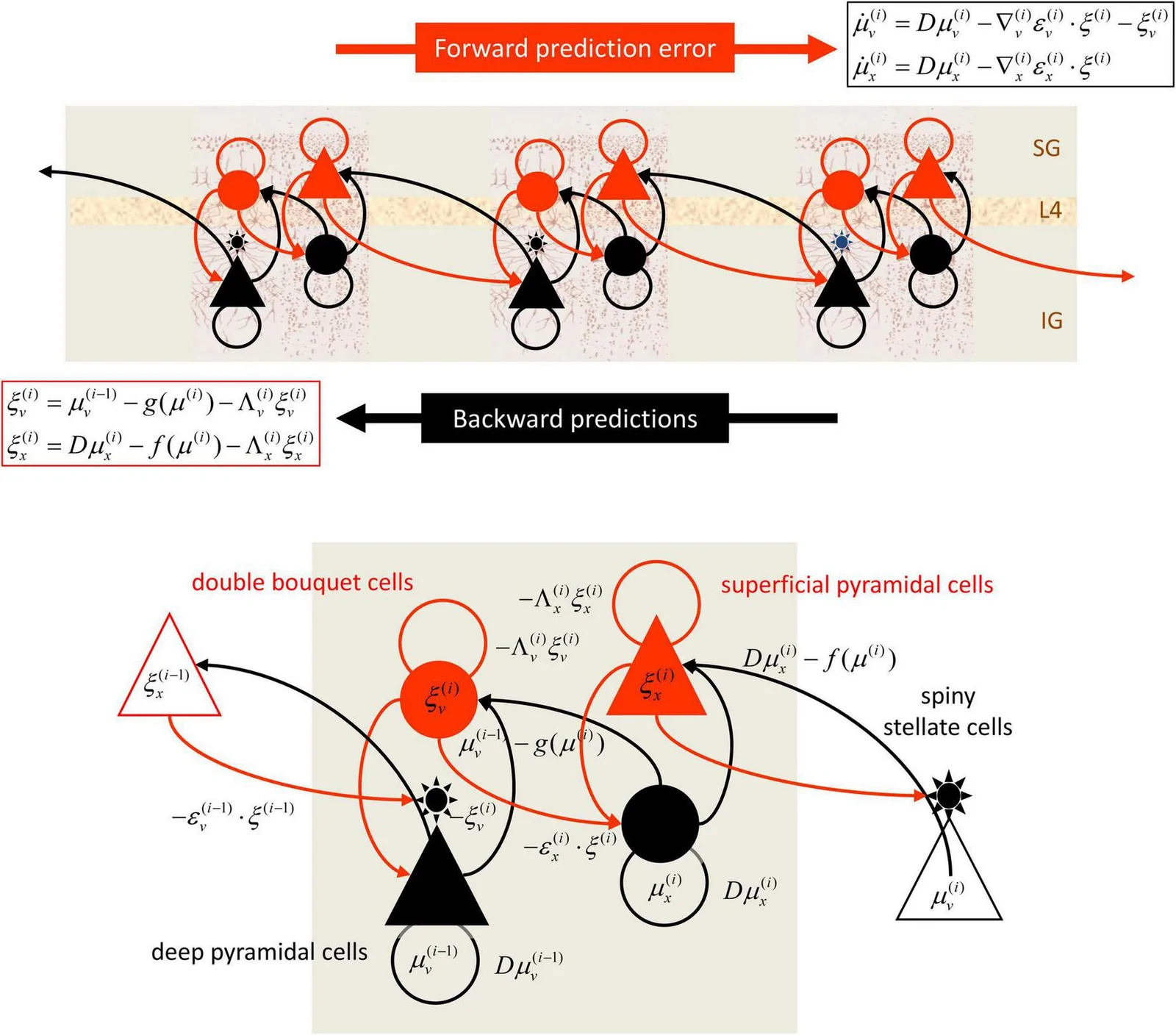
Canonical architectures for predictive coding: this schematic illustrates the neuronal circuits thought to encode probabilistic representations of hidden states, under a hierarchical generative model. It shows the speculative cells of origin of forward driving connections that broadcast prediction errors from a lower area to a higher area and the backward connections that are used to convey predictions. These predictions try to explain away input from lower areas by resolving prediction error. In this scheme, the sources of forward connections are superficial pyramidal cell populations and the sources of backward connections are deep pyramidal cell populations. The neuronal populations representing hidden states and causes are in black and populations broadcasting prediction errors in red. The lower panel provides the functional form of a generalized predictive coding scheme that we will refer to later, with hidden causes (*v*) on the left and hidden states (*x*) on the right (hidden *causes* link successive levels, while hidden *states* link successive time points). The variables relate to the generalized predictive coding scheme detailed in Friston K. (2008). The equations in the red box show how precision-weighted prediction errors depend upon expected causes and states, while the equations in the black box show how expected causes and states depend upon prediction errors. In this and subsequent figures, precision weighted prediction errors are denoted by ξ = Πε = ε−Λξ, were Π is a precision matrix and Λ can be regarded as the strength of horizontal connections (Pinotsis et al., 2014; Tang et al., 2023). In this, and subsequent figures, all the variables are taken to be in generalized coordinates of motion, such that *D* returns generalized motion: please see Friston K. (2008) from which this figure is adapted, under a CC BY-NC license. SG, L4 and IG denote supragranular, granular or layer IV, and infragranular, respectively.

BOX 1 **Neurobiological mechanisms of predictive coding**

Throughout this review, we touch upon the mechanistic implications of predictive coding, and its generalizations, for neurobiology. This box offers a summary of the ways in which this theoretical stance makes sense of findings from empirical neuroscience.

– The hierarchical organization of cortical areas as evidenced by the asymmetry of laminar connectivity patterns for forward and backward projections (Zeki and Shipp, 1988), and associated cell populations (Mumford, 1992), follows from the separation into prediction and prediction error units.

– The difference in spectral properties (Mejias et al., 2016; Michalareas et al., 2016) in the transmission of ascending and descending signals follows from the nonlinear (frequency multiplying) and linear mixing of ascending prediction errors and descending predictions, respectively (Friston, 2019).

– The dynamics resulting from recurrent connectivity are intrinsically stable (Crick and Koch, 1998) in that they suppress prediction error.

– Activity-dependent plasticity (Bliss and Lomo, 1973) and neuromodulation are emergent consequences of the mean-field effects implied by the factorisation shown in Figure 3.

– Different kinds of precision appear to be mediated, in part, by distinct neurotransmitters (Gu, 2002; Yu and Dayan, 2005; Parr and Friston, 2017), with increasing evidence (Marshall et al., 2016) that prior precision depends upon noradrenergic (Sales et al., 2019; Vincent et al., 2019), sensory precision on cholinergic (Moran et al., 2013), and action selection on dopaminergic (Schwartenbeck et al., 2015a) signalling. – The actions of these neurochemicals, in weighting prediction errors, may be through lateral connectivity and modulation of postsynaptic hyperpolarisation (Bröcher et al., 1992).

Mumford and colleagues (Lee and Mumford, 2003) pursued these ideas using particle filters and Bayesian belief propagation, which we will later see can be read as a form of predictive coding. In the interim, Rao and Ballard wrote their seminal paper on predictive coding in the visual cortex (Rao and Ballard, 1999). This, and a companion treatment (Rao, 1999), were important for several reasons. One, possibly underappreciated, reason was the distinction between optimizing representations of external (a.k.a., hidden) states causing observations – i.e., *inference* – from the parameters encoding the underlying cause-effect structure – i.e., *learning*. In short, inference and learning were both explainable in terms of minimizing prediction error. The second contribution was to establish the equivalence between predictive coding and Kalman filtering. This meant predictive coding could be read as a special case of Bayesian filtering (Rao, 1999; Kschischang et al., 2001; Lee and Mumford, 2003; Mitter and Newton, 2003); namely, sequential data assimilation under a state-space generative model. This extended the compass of predictive coding to include the Bayesian brain hypothesis (Knill and Pouget, 2004) and, indeed, motor control (Knill and Pouget, 2004; Körding and Wolpert, 2004). The latter link was gracefully provided by a formal equivalence between Kalman filtering and linear quadratic control, as noted by Kalman himself (Todorov and Jordan, 2002; Todorov, 2008).

Retrospectively, the Bayesian filtering perspective is compelling, when one appreciates that an idealized particle filter is an extended Kalman or variational filter (Friston K. J., 2008; Friston et al., 2008). Intuitively, particle filtering (and related procedures like unscented filtering (Schiff and Sauer, 2008)) use realizations of lots of little predictive coding schemes to obtain an ensemble estimate. However, if one assumes that one has an infinite number of particles, the ensuing probability density dynamics can be written as a variational Bayesian filter (Friston K. J., 2008), which just is predictive coding.

Around the turn of the century, the Bayesian brain hypothesis started to attract more attention (Knill and Pouget, 2004; Ma et al., 2006); echoing – in one sense – earlier ideas of perception as hypothesis testing and analysis by synthesis (Gregory, 1968, 1980; Yuille and Kersten, 2006). These ideas themselves spoke to Helmholtz’s notion of unconscious inference (Helmholtz, 1860/1962, 1878/1971) nicely summarized as:

“*Objects are always imagined as being present in the field of vision as would have to be there in order to produce the same impression on the nervous mechanism*”.

Indeed, Peter Dayan and Geoffrey Hinton created a Helmholtz machine (Dayan et al., 1995) predicated on variational free energy – introduced by Bogolubov (1966) and used by Feynman (Feynman, 1955, 1972) – to convert an intractable (path integral) inference problem into a tractable (computable) optimisation problem. In short, variational free energy bounds the likelihood of some data under a model of how those data were caused, such that minimizing free energy implicitly identifies the best explanation for those data (c.f., Inference to the best explanation). So, was the Helmholtz machine a predictive coding machine in disguise? Probably not. The Helmholtz machine and variants – such as modern day variational autoencoders (Marino, 2021) – came from machine learning. The emphasis here is on *learning*, as opposed to *inference* or state-estimation afforded by Bayesian (e.g., particle) filters (Beal, 2003; Winn and Bishop, 2005). If we roll forward a few decades, these early Bayesian learning machines are now seen as *learning to infer*, sometimes referred to as *amortizing* inference (Zhang et al., 2019; Mazzaglia et al., 2022). In other words, inference optimizes beliefs about the causes of some data on a case-by-case basis. Amortization learns a direct mapping from data to beliefs from multiple cases.

Inference and learning are best described in relation to a *generative model*. Any scheme that explains or predicts (sensory) data must entail a generative model: i.e., a probabilistic model that can generate the observable (sensed) consequences of unobservable (hidden) causes. The generative model covers different kinds of hidden (a.k.a., random) variables; such as states of the world that are hidden behind the sensory apparatus (e.g., sensory epithelia for the skull-bound brain). These include the *states*, *parameters* and *structure* of the generative model, which have to be *inferred*, *learned* and *selected*, respectively. In order to optimize or estimate these unknowns, it is necessary to encode the uncertainty inherent in those estimates, so that uncertainty about one set of unknowns informs the estimates of others.

## 3 The importance of being precise: neuromodulation and representational sharpening

Within inference, one can distinguish between the expected state of the world and the uncertainty associated with that estimate. This uncertainty – or its complement, precision – equips predictive coding with an inferential aspect; much in the same way that a statistician measures some effect size in relation to its standard error. The requisite estimate of confidence or credibility is often referred to as *precision*. Mathematically, it is easier to deal with precision than its complement such as uncertainty (Knill and Pouget, 2004; Strange et al., 2005; Yu and Dayan, 2005), variance (Harville, 1974; Dempster et al., 1981), entropy (Klein and Meijer, 1954; Jaynes, 1980), volatility (Mathys et al., 2011; Palmer et al., 2017), *et cetera*. One reason to think in terms of precision inherits from its role in predictive coding as a rate constant or *learning rate*. We will return to this later when considering reward prediction errors. At present, the separation of various unknowns – into expectations, precisions and parameters – lends itself to a graceful interpretation in terms of perceptual inference, attention and learning as illustrated in Figure 3.

**FIGURE 3:**
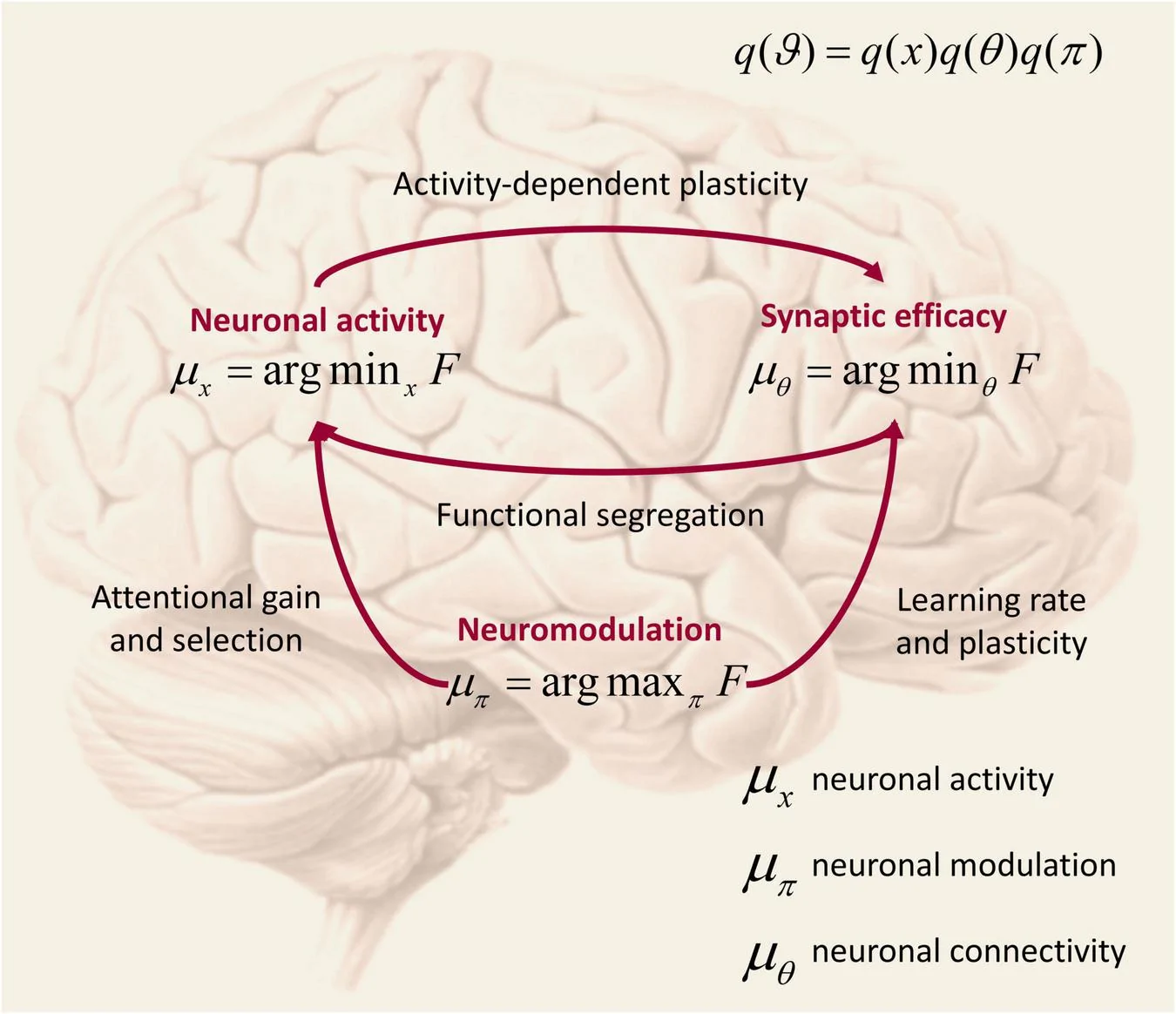
Carving neurobiology at its joints: this schematic illustrates the various neurobiological processes entailed by separating estimates of hidden variables into three conditionally independent sets. Technically, this separation corresponds to a mean field factorization (see top equation). This equation expresses a probability density over everything, in terms of a (mean-field) factorization, where each factor is encoded in terms of the sufficient statistics of its marginals. These statistics (*e.g*., mean or expectation) change to minimize variational free energy to provide Bayes optimal estimates. This Figure uses *neuronal activity*, *modulation* and *connectivity* to encode *hidden states*, *precision* and *parameters*, respectively. Crucially, the optimization of any one set depends on the others. On this view, one can regard changes in neuronal activity as encoding expected states in a way that is informed by – or dependent on – precision and parameters. In other words, synaptic efficacy is determined by afferent connectivity and neuromodulation of postsynaptic gain. Similarly, learning can be read as updating synaptic connections in a way that depends upon neuronal activity and neuromodulation. Adapted from Friston K. (2008) under a CC BY-NC license.

The separation in Figure 3 was one of the key commitments of an early formulation (Friston, 2005) of cortical responses – predicated on predictive coding – that would later form the basis of the free energy principle (Friston, 2010). In brief, not only did one have to estimate the latent states and parameters – under a given model structure – one also had to estimate the confidence, credibility or *precision* afforded those estimates. This was important because it gave rise to different strands of predictive coding research and, arguably, a divergence of research agendas.

From the perspective of engineering, precision underwrites the Kalman gain applied to prediction errors during the update step of a Kalman filter. The Kalman gain is effectively the ratio of precisions afforded sensory prediction errors relative to state prediction errors, under a generative model. [A *generative model* is just a joint probability distribution over unobservable causes and observable consequences. It is generally decomposed into the *likelihood* of consequences, given their causes, and *a prior* over causes]. In predictive coding, precisions are associated with expected squared prediction errors because the prediction errors score the amplitude of random fluctuations, which have the attribute of precision. If the sensory precision or Kalman gain is high, then sensory prediction errors are afforded more weight and are thereby preferentially selected for updating estimates of latent states: see Figure 2. This means the implicit Bayesian belief updating rests upon the precision of prior beliefs (i.e., state noise), relative to the precision of sensory observations (i.e., observation noise).

This important aspect of predictive coding – and its context sensitivity – is largely ignored in some treatments; e.g., (Heeger, 2017; Keller and Mrsic-Flogel, 2018). Other disciplines have focused almost exclusively on precision (Shipp, 2016); particularly, in cognitive neuroscience and computational psychiatry; e.g., (Kok et al., 2012b; Adams et al., 2013b; Brown et al., 2013; FitzGerald et al., 2015; Ainley et al., 2016; Yon and Frith, 2021). Key applications in cognitive neuroscience associate the optimization of precision with attentional selection (and sensory attenuation) (Brown et al., 2013; Pareés et al., 2014; Limanowski, 2017), offering a formal account of things like representational sharpening and biased competition (Spratling, 2008; Feldman and Friston, 2010). See Figure 4. In computational psychiatry, aberrant precision weighting or (postsynaptic) gain control is a dominant theme in understanding psychopathology in terms of false inference (Edwards et al., 2012; Hohwy, 2012; Kok et al., 2012b; Paton et al., 2012; Lawson et al., 2014; FitzGerald et al., 2015; Ainley et al., 2016; Kiverstein et al., 2019; Smith et al., 2020; Yon and Frith, 2021; Haarsma et al., 2022): see references in (Friston, 2022). See Box 2. Interestingly, hierarchical (c.f., metacognitive) precision weighting has also been a focus in the emerging field of neurophenomenology (Limanowski, 2017; Limanowski and Friston, 2018; Sandved-Smith et al., 2021).

**FIGURE 4:**
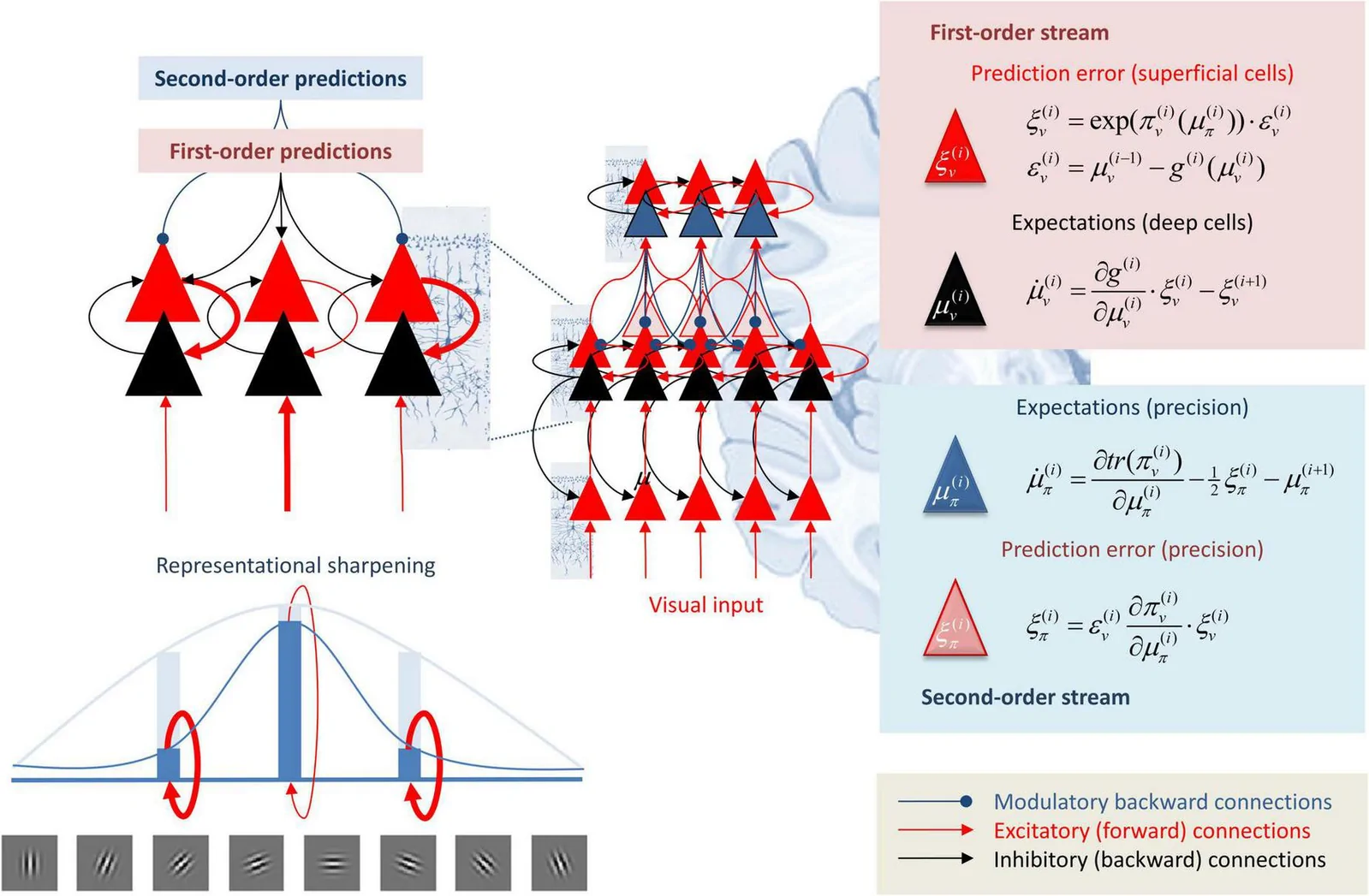
Predictive coding and precision engineering: This schematic describes the hierarchical message passing implicit in predictive coding based on deep generative models. In this scheme, sensory input is conveyed to sensory (e.g., primary visual) cortex via ascending prediction errors (e.g., from the lateral geniculate). Posterior expectations, encoded by the activity of deep pyramidal cells, are driven by ascending prediction errors (red arrows). These cells then provide descending predictions (black arrows) that elaborate prediction errors at the lower level. At the same time, they are subject to lateral interactions that mediate (empirical) priors. Crucially, prediction errors are modulated by *predictions of their precision* (blue arrows). The predicted precision is based on the sum of squared prediction errors. This means we have two sets of ascending and descending counter streams: the first dealing with predictions of (first-order) *content* and the second dealing with (second-order) *context*; namely, the precision of first-order prediction errors. Heuristically, expectations about precision release posterior expectations from constraints in the vicinity of an inferred attribute or trajectory, and allow them to respond more sensitively to ascending input. This is illustrated on the lower left (Representational sharpening). The key point here is that prediction errors compete for influence over pyramidal cells representing stimulus features (i.e., expectations). If a representation is released from top-down constraints, it is disinhibited and becomes more sensitive to ascending prediction error. Conversely, if a particular prediction error is afforded greater top-down precision, it effectively pulls the posterior expectation toward its prior mean of zero; as illustrated by the red arrows in the lower left panel. In this example, the activity of the middle deep pyramidal cell (black triangle on the upper left) could encode the expected orientation of a local stimulus (indicated by the Gabor patches on the lower left). In terms of extra-classical receptive field effects, this corresponds to representational sharpening (Kok et al., 2012a). For a more detailed description of the implicit belief updating, equations, and accompanying neuronal dynamics, see Kanai et al. (2015). Adapted from Friston (2018) under a CC BY-NC license.

To contextualize the work in these fields, we have to consider two generalizations of predictive coding. Thus far, we have a formulation of predictive coding as a particular instance of Bayesian filtering or, technically, approximate Bayesian inference. Approximate Bayesian inference refers to the optimization of a variational bound on the evidence for the requisite generative model used to compress or represent sensory data. This is the variational free energy due to Feynman (Feynman, 1972; Dayan et al., 1995; Beal, 2003; Winn and Bishop, 2005) that rests upon specifying a functional form for posterior beliefs (usually a Gaussian form, leading to a generalization of the Laplace assumption). See Gottwald and Braun (2020) for a discussion of free energy in this setting.

Technically, variational free energy converts an impossible marginalization problem – for any inference – into a tractable optimisation problem, where one minimizes variational free energy and thereby maximizes model evidence (a.k.a., marginal likelihood) to within a bound approximation. In machine learning, negative variational free energy is known as an evidence lower bound or ELBO (Winn and Bishop, 2005). The details are not important here, other than to appreciate that (precision-weighted) prediction errors – that define predictive coding – are simply the *gradients of variational free energy* with respect to the unknown quantities (Tschacher and Haken, 2007). However, this is only true under the Laplace assumption, which assumes that all random fluctuations are well-behaved; i.e., are drawn from a Gaussian distribution. While this seems a severe criterion, note that almost all random fluctuations in continuous space are Gaussian up to a quadratic approximation of their (log) probability densities around a mode. With this simple assumption, one can then write down a predictive coding or Bayesian filtering scheme for any generative model and minimize variational free energy with respect to the unknown states, parameters and structure. This variational formulation provides the calculus that grandfathers any implementation of the Bayesian brain under Gaussian assumptions. On this view, one could define predictive coding as the variational inversion of a continuous state-space model under Gaussian assumptions about random fluctuations. Is this a sufficient definition?

Not quite. One has to ask two things of the generative model. First, what is the order of the dynamics? In other words, much like the brain encodes position and velocity (and higher of orders of motion), one can ask how many orders of motion are implicit in the state-space models used for predictive coding. The answer – for a vanilla Kalman filtering scheme – is two orders of motion; namely, position and velocity. But why stop there? This leads to the first generalization of hierarchical predictive coding to accommodate high order dynamics, such as acceleration and jerk. This in turn leads to *generalized predictive coding* or filtering (Kerr and Graham, 2000; Friston et al., 2008, 2010b) that becomes especially important for the second generalization; namely, active filtering or inference; e.g., (Friston, 2011; Friston et al., 2011; Perrinet et al., 2014).

## 4 From unconscious inference to active inference

This generalization reflects the enactive turn at the beginning of the century and asks, could one deploy predictive coding in the setting of motor control? In one sense – following Todorov and colleagues (Todorov, 2008; Kappen et al., 2012) – we already know the answer: one simply uses a predictive coding scheme to control the motor plant (Adams et al., 2013a). In engineering, this is commonplace, and is variously known as path-integral control (Braun et al., 2011; Kappen et al., 2012), model predictive control (Doya, 1999; McKinstry et al., 2006; Schwenzer et al., 2021; Taniguchi et al., 2023) or control as inference (Tassa et al., 2011; Baltieri and Buckley, 2018; Fields et al., 2023). Indeed, in neurobiology this perspective emerges in perceptual control theory (Mansell, 2011) and the equilibrium point hypothesis (Feldman, 2009) – see (Parr et al., 2025b) for a recent discussion of these schemes in relation to active inference. Interestingly, one of the key architects of predictive coding in the brain has now turned to active inference and sensing, in terms of *active predictive coding* (Rao et al., 2023).

We now arrive at a generalization of predictive coding – under continuous state-space models – that can be summarized as the minimization of variational free energy under the Laplace assumption through both perception and action. The active element is facilitated by the use of higher orders of motion, as we now have an opportunity to resolve sensory prediction errors via the gradients of sensory input with respect to action – which depend upon higher orders of motion affecting hidden states, e.g., in a Newtonian world one acts through inducing accelerations by applying forces.

BOX 2 **Translational implications of predictive coding**

Any theory that improves our understanding of the brain should also facilitate our understanding of, and ultimately treatment of, clinical syndromes in psychiatry and neurology. The usefulness of predictive coding, and its generalizations, here rest upon a set of results known as the Complete Class Theorems (Wald, 1947). These have been widely interpreted as saying that any behavior (Daunizeau et al., 2010), including those that might be seen as pathological, is Bayes optimal under some set of prior beliefs (Parr et al., 2018b). From the perspective of predictive coding, this implies one should not seek to understand the way in which a patient’s behavior is suboptimal, but to understand the set of predictions they must be making for that same behavior to appear optimal (Schwartenbeck et al., 2015c). Pursuing this reframing, we arrive at three applications. First is a means of phenotyping, which might help to target treatments more effectively (c.f., advances in cancer therapies driven by molecular phenotyping). Second is that the parameters used for these phenotypes are associated with neural mechanisms (see Box 1) that afford potential therapeutic targets. Third is the opportunity to optimize a patient’s environment to make it easier for that person to resolve troublesome prediction errors. For instance, one could offer clear rhythmic cues to help alleviate imprecise temporal predictions in Parkinson’s disease or design educational settings that minimize sensory noise to mitigate failures of sensory attenuation; e.g., in those with autism.

The following is a non-exhaustive list of conditions in which the above ideas have been used in psychiatry and neurology:

– Primary psychotic conditions, including schizophrenia (Adams et al., 2013b,^2015^, ^2021^; Benrimoh et al., 2018, 2019; Corlett et al., 2019)

– Developmental conditions, including autism (Lawson et al., 2014, 2021; Palmer et al., 2017)

– Affective conditions, including depression (Kube et al., 2020), anxiety (Harris et al., 2023; Bae, 2025), and post-traumatic disorders (Linson et al., 2020)

– Compulsive behaviors including both addiction (Schwartenbeck et al., 2015c) and obsessive-compulsive disorder (Fradkin et al., 2020)

– Movement disorders (Parr et al., 2021), including pyramidal, cerebellar, and extrapyramidal syndromes (Parr et al., 2025a)

– Visuospatial disorders including visual neglect (Parr and Friston, 2017) and hallucinatory phenomena in synucleinopathies (Parr et al., 2018a,Collerton et al., 2023, 2024)

– Cognitive features of dementia syndromes, including apathy (Hezemans et al., 2020) and memory impairment (Parr et al., 2026)

– Disorders of language, including aphasia (Sajid et al., 2021) and dysfluency syndromes (Usler, 2025)

– Functional neurological disorders (Edwards et al., 2012; Pareés et al., 2014)

Predictive coding, so defined, can handle difficult problems that entail nonlinear, hierarchical models with separation of temporal scales. See ref (Friston and Kiebel, 2009) for an example of recognizing structured speech or syntax, under the right kind of generative model. Figure 5 provides an example of the same generalized predictive coding scheme that was used to simulate handwriting. It is at this point we can now turn to predictive processing.

**FIGURE 5:**
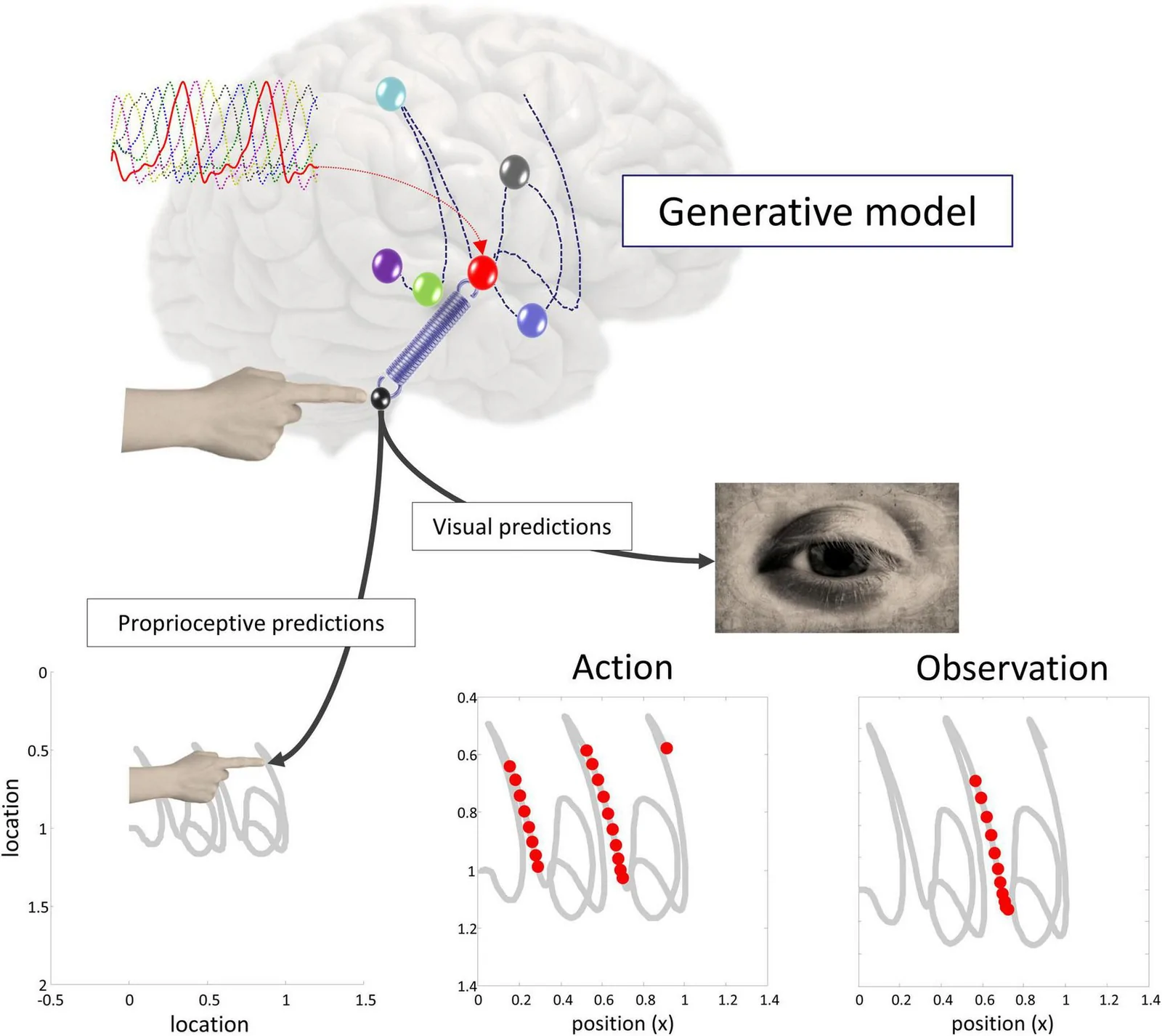
Action and its observation. This Figure illustrates simulations of active inference – here, writing – in terms of conditional expectations about hidden states of the world, consequent predictions about sensory input and ensuing action. The dynamics that underwrite this behavior rest upon prior expectations based upon Lotka-Volterra dynamics: these are the six colored lines in the upper left inset. In this generative model, each state is associated with a location in extrapersonal space that attracts an agent’s finger. In effect, inferred states supply predictions of what proprioceptive states should register if the agent’s beliefs were true. Action then resolves the ensuing proprioceptive prediction error by fulfilling predicted changes in angular velocity, through exerting forces on the agent’s joints (not shown). The subsequent movement of the arm is traced out in the lower left panel. This trajectory is plotted in a moving frame of reference, so that it looks like handwriting (e.g., a succession of ‘j’ and ‘a’ letters). The lower left panels show the activity of the fourth neuronal state under ‘action’, and ‘action-observation’. During action, sensory states register the visual and proprioceptive consequences of movement, while – under action observation – only visual sensations are available; as if the agent was watching another agent. The red dots correspond to the times during which this state exceeded an arbitrary threshold. They key thing to note here is that this neuronal state responds preferentially when, and only when, the motor trajectory produces a down-stroke, but not an up-stroke, evincing a cardinal feature of neuronal responses, namely, place cell activity and directional selectivity. Furthermore, with a slight delay, this internal state responds during action and action observation. From a neurobiological perspective, this speaks to mirror neuron activity (Gallese and Goldman, 1998; Rizzolatti and Craighero, 2004; Kilner et al., 2007). This is an interesting illustration of predictive coding in action because the proprioceptive and visual signals are generated by the agent under its own prior beliefs. In short, the agent is authoring its own sensorium – in the words of Andy Clark – “predicting itself into existence”. From a motor control perspective, descending proprioceptive predictions play the role of motor commands that are fulfilled by classical motor reflexes, while descending exteroceptive predictions play the role of corollary discharge (Bays and Wolpert, 2007; Feldman, 2009). Please see Friston (2011) for further details.

## 5 From predictive coding to predictive processing

Predictive processing was introduced in Andy Clark’s seminal 2013 treatment of the predictive brain (Clark, 2013). One might ask why Andy chose *predictive processing*, which has enjoyed enormous uptake in the philosophy and cognitive neuroscience literature. The reason was subtle but compelling. He did not want to limit the scope of prediction to mere predictive coding formulations that ignored active sensing of the kind emphasized by Helmholtz (1878/1971). See also (Friston et al., 2012). For example:

“*Each movement we make by which we alter the appearance of objects should be thought of as an experiment designed to test whether we have understood correctly the invariant relations of the phenomena before us, that is, their existence in definite spatial relations*.” ‘The Facts of Perception’ (1878) in The Selected Writings of Hermann von Helmholtz, Ed. R. Karl, Middletown: Wesleyan University Press, 1971 p. 384

On the other hand, he did not want to burden predictive processing with the broader commitments of the free energy principle based upon Feynman’s variational principle (Friston, 2010), which was emerging at that time. He therefore focused on predictive coding in an enactive and embodied setting (Clark, 2017, 2023). Furthermore, subsequent work quickly highlighted the importance of precision (Hohwy, 2012, 2013, 2016, 2025; Clark, 2016), such that predictive processing became synonymous with the minimisation of *precision-weighted prediction errors*. This was important because it invites cognitive neuroscientists – interested in attention and salience – to engage with predictive processing accounts: e.g., (Hohwy, 2012; Kok et al., 2012b; Paton et al., 2012; Jiang et al., 2013; Auksztulewicz and Friston, 2015; Vossel et al., 2015; Ainley et al., 2016; Heilbron and Chait, 2018; Parr and Friston, 2019; Limanowski, 2022).

On a philosophical note, the equivalence between minimizing precision-weighted prediction errors, minimizing variational free energy and maximizing model evidence leads to the nice notion of self-evidencing (Hohwy, 2016). In other words, predictive coding may be the mechanism by which we gather or accumulate evidence (Gold and Shadlen, 2003; Hesselmann et al., 2010) for our models of the lived world.

In a translational context, it also speaks to the importance of predictive coding for explaining psychopathology. The story here is fairly straightforward: If psychology can be cast as inference to the best explanation (Seth, 2015), then psychopathology can be cast as false inference. Here, false inference can be read in its simplest sense of type I and II errors. Type I errors are when one infers something is there when it is not (e.g., hallucinations and delusions). Conversely, type II errors involve inferring something is not there when it is (e.g., neglect syndromes and dissociative disorders). As noted above, this led to a whole literature in computational psychiatry; in which many neurological and psychiatric conditions (see Box 2) were cast in terms of aberrant precision weighting: e.g., (Adams et al., 2013b; Lawson et al., 2014; Gu et al., 2015; Sterzer et al., 2018; Kiverstein et al., 2019; Rae et al., 2019; Lyndon and Corlett, 2020). One interesting (aberrant salience) story (Kapur, 2003) rests on the notion of reward prediction errors.

## 6 Predictive coding in reward learning

With the emergence of predictive coding as a scheme for belief-updating in the brain, progress was being made in the field of reinforcement learning (Schultz et al., 1997; Schultz, 1998; Daw and Touretzky, 2002; Doya, 2002; Daw and Doya, 2006; Pessiglione et al., 2006). Much of this progress turned upon the notion that dopamine encoded a *reward prediction error*. This was perhaps a little unfortunate, because it gave the specious impression that dopamine was encoding a prediction error in the spirit of predictive coding. However, when applying the principles of predictive coding to decision-making – in the sense of planning as inference (Friston et al., 2013) – it became evident that dopamine was not encoding prediction errors *per se* but the precision of inferences about choices or plans (Schwartenbeck et al., 2015a,b). Intuitively, this would look much like a reward prediction error in some circumstances, as achieving an unexpected reward might increase one’s confidence in the current course of action. In contrast, an outcome that is more aversive (or less rewarding) than anticipated, might decrease one’s confidence that they are pursuing a likely course of action, thereby signaling what looks like a negative prediction error. To this extent, the precision account of dopamine makes the same empirical predictions as the reward prediction error account. However, one can go beyond this. If phasic dopamine signals changes in policy precision, then one would expect similar dopaminergic responses to (non-rewarding) information that informs choice behavior. This is compatible with the literature reviewed in (Horvitz, 2000) in which (non-rewarding) cues – that impose constraints upon the likely course of action – can themselves trigger dopamine release.

An important distinction between dopamine, as encoding reward prediction error versus policy precision, rests on the fact that reward prediction error formulations inherit from reinforcement learning, while active inference encompass both inference and learning. For example – in the setting of hypodopaminergic clinical states – the difference between a bradykinetic presentation of Parkinson’s disease, before and after dopaminergic medication, is evident before any learning can occur (Lees, 2010). This clinical observation lies outside the explanatory scope of reward-learning but is neatly accommodated in a precision-based account under which imprecise motor planning results in impoverished movement.

Subsequent interpretations of dopaminergic responses focused on the implicit encoding of precision, novelty and related quantities (Bunzeck and Düzel, 2006; Wittmann et al., 2008; Bromberg-Martin and Hikosaka, 2009; Hazy et al., 2010; Barto et al., 2013; Iglesias et al., 2013; Schwartenbeck et al., 2013; Starkweather et al., 2017; Haarsma et al., 2018). This speaks to an important link between precision, Kalman gain and learning rate.

Recall from above that the relative precision of priors and sensory evidence determines the Kalman gain and thereby, the rate of belief updating. A simple dimensional analysis of precision – under any state-space model – shows that precision has the units of per time and therefore scores the plasticity or learning rate in any scheme that can read as predictive coding. On this view, dopamine acts as a learning rate (Niv et al., 2005; Rouhani and Niv, 2021), consistent with its role in working memory and gain control (Kojima and Goldman-Rakic, 1982; Cohen and Servan-Schreiber, 1992; Goldman-Rakic et al., 1992; Goldman-Rakic, 1997; Braver et al., 1999).

In predictive coding terms, this suggests certain dopaminergic functions – e.g., of D_1_ receptors in prefrontal cortex (Sawaguchi and Goldman-Rakic, 1991) – are to mediate the *precision-weighting* of precision-weighted prediction errors. This view of dopaminergic function admits a plurality of functional roles (Schultz, 2007), depending upon where precision-weighting is mediated (e.g., mesocortical, mesolimbic, nigrostriatal, *et cetera*); thereby linking its role in working memory (Sawaguchi and Goldman-Rakic, 1991), planning (Kakade and Dayan, 2002; Niv et al., 2005), addiction (Redish, 2004), Parkinson’s disease (Rutledge et al., 2009), schizophrenia (Cohen and Servan-Schreiber, 1992) and so on. These roles can be formulated – under predictive coding – in terms of aberrant salience (Berridge and Robinson, 1998), cortical gain control (Abbott et al., 1997), and excitation-inhibition balance (Nitsche et al., 2010; Lisman, 2012), and concomitant failures of selective attention and sensory attenuation (Shergill et al., 2005; Adams et al., 2013b; Brown et al., 2013).

## 7 Predictive coding in machine learning

In a further turn of affairs, the machine learning community [re]turned to predictive coding in the past decade, with an interesting twist. In brief, the kind of predictive coding one finds in this literature focuses on the problems encountered with back propagation of errors: e.g., vanishing gradients (Hochreiter and Schmidhuber, 1997), overfitting (Sengupta and Friston, 2018), and so on. The key thing this field takes from predictive coding is the local (variational free) energy minimisation at each level in a deep (hierarchical) generative model: c.f., (Scellier and Bengio, 2017). This means, in principle, predictive coding offers an alternative to back-propagation of errors that, in principle, should be more efficient than current machine learning procedures (Whittington and Bogacz, 2017; Millidge et al., 2022a,b; Salvatori et al., 2021, 2022).

The twist here is that instead of optimizing the structure and functional form of the generative model, machine learning variants apply the principles of predictive coding to a standard neural network architecture. Although there is current excitement about the potential of predictive coding in this setting, it has yet to be established at scale. From the perspective of predictive coding, the focus on learning the parameters (i.e., weights) of a neural network means these applications are largely limited to static models (that preclude dynamics). Another issue – with this application of predictive coding – is a failure to encode uncertainty about the model parameters, which means computation of the requisite variational free energy gradients (or fixed-point solutions) is compromised. This follows because predictive coding – in its generalized form – requires probability densities over all random variables, including model parameters: c.f., Figure 3.

Having said this, there are interesting developments in connecting transformer architectures to Bayesian filtering, where attention heads can be cast as estimates of precision or Kalman gain (Singh and Buckley, 2023). This speaks to a nice convergence of machine learning and computational neuroscience on the mechanisms of attentional selection (Feldman and Friston, 2010; Vaswani et al., 2017; Mirza et al., 2019). More generally, it completes the convergence of predictive coding (Elias, 1955b) and Bayesian filtering under Feynman’s variational principle of least action (Feynman, 1955); in other words, there is a general account of predictive processing that simply invokes the minimisation of a variational bound on the evidence for generative models; namely, the free energy principle.

## 8 A reverse history of predictive coding: neurobiology from first principles

In retrospect, we can now review the history of predictive coding through its generalizations. Retrospectively, the starting point is the free energy principle, which points to predictive coding as a universal description of any open (brain-like) system that self-organizes to some characteristic states. The mathematical details are not important here. The key thing to take away is that any (multidimensional random dynamical) system – from a postsynaptic process (Kiebel and Friston, 2011) to a person (Hohwy, 2016) – can be described as minimizing its variational free energy (Friston et al., 2023). This description rests upon associating the internal states (e.g., neuronal activity and synaptic efficacy) with the parameters of some Bayesian beliefs about the external causes of sensory inputs (including the system’s own actions). If we make the further assumption that the random fluctuations – inherent in any random dynamical system – are (by central limit theorem) Gaussian, then we have predictive coding. In short, anything that exists in the sense of possessing characteristic states can be described as if it is engaged in predictive coding (Friston et al., 2014; Friston et al., 2023): see Figure 6.

**FIGURE 6:**
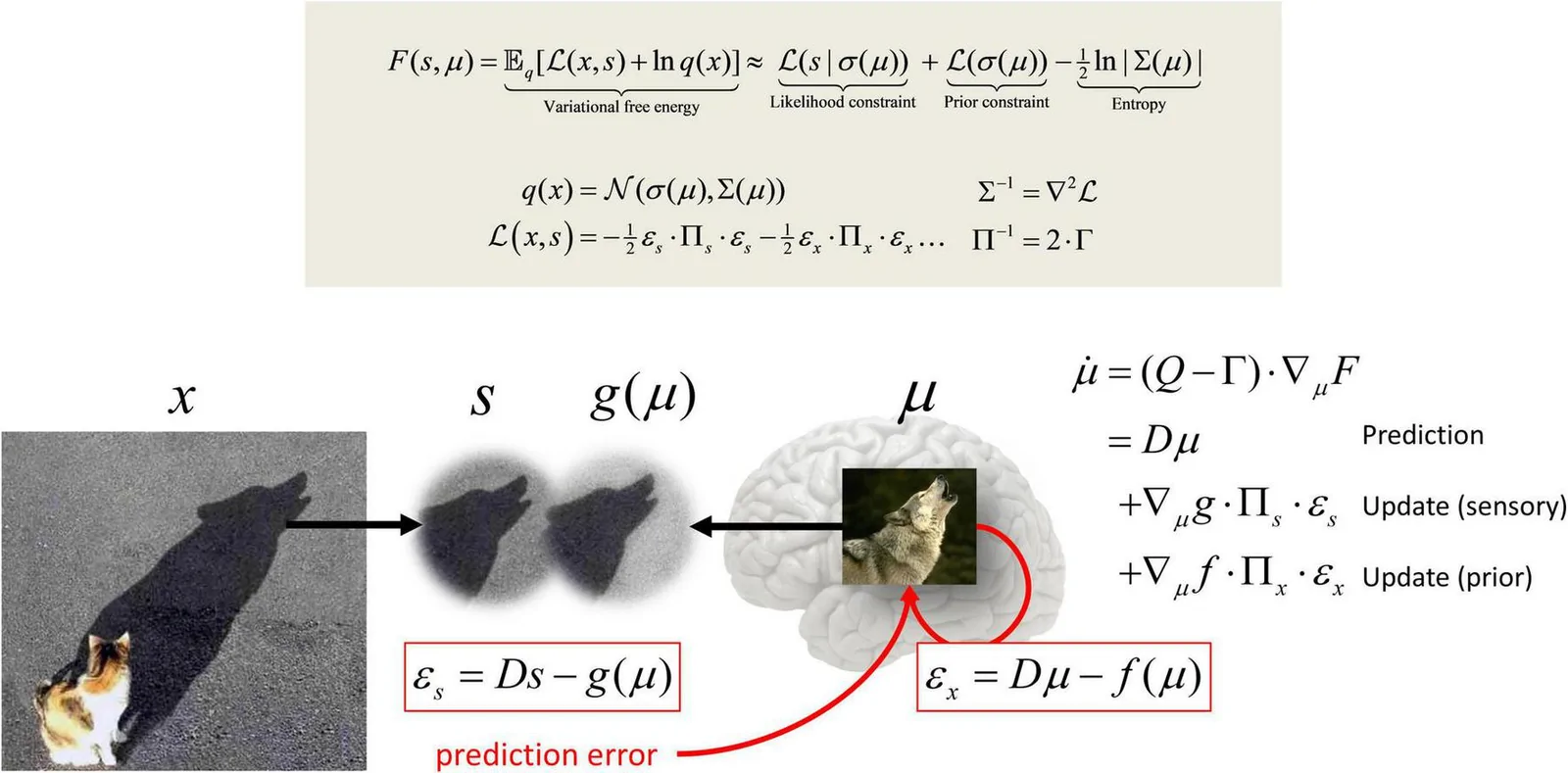
Bayesian filtering and predictive coding: this graphic illustrates the formal equivalence between predictive coding and the self-organization of internal states of any (random dynamical) system to which the free energy principle applies. The equations above the graphics show that one can rearrange the dynamics of internal states (μ) – expressed as a gradient flow on variational free energy (*F*) – in the form of a Bayesian filter; comprising prediction and update terms. Heuristically, the prediction inherits from the conservative or non-dissipative (*Q*) part of the dynamics, while the update term is a dissipative (Γ) gradient flow on variational free energy (Bhatia et al., 2013). The free energy gradients can always be expressed as a prediction error, where the predictions rest upon associating the internal states with the sufficient statistics of (Bayesian) beliefs about external states. Intuitively, the functional form of the ensuing dynamics – technically, paths of least action – can be read as follows: if one had a (Bayesian) belief or expectation about the causes of sensory impressions (here, a howling dog), then one can predict what one would see, given a model (*g*) that generates predictions of what would be observed, given that expectation. By subtracting the predictions from the sensed input (*s*), we have a prediction error (ε). The upper equations then imply that prediction errors – suitably weighted by precision and nonlinearities in the generative model – drive internal states to provide a better explanation of the sensory impression, until prediction error is minimized and the free energy gradients are destroyed (Haken and Portugali, 2021). Note that one will never know the true cause of sensory observations, because inference to the best explanation simply minimizes precision-weighted prediction errors (i.e., variational free energy). In this example, the actual cause was a cat.

This move elevates predictive coding from some ad hoc process theory to a first principles account; or at least an application of first principles [see Hohwy (2020) for a detailed analysis of this distinction]. This is important because questions like “what is the evidence for predictive coding?” can be reframed as “how is predictive coding instantiated in this brain?”. In other words, key questions are less about whether predictive coding is an apt description; they are about how predictive coding is implemented mechanistically-and under which generative model-in a substrate-dependent fashion (Heilbron and Chait, 2018, Walsh et al., 2020, Hodson et al., 2024). This is sometimes discussed in terms of mortal computation (Hinton, 2022, Ororbia and Friston, 2023). Furthermore, the same questions can be asked at multiple scales; ranging from cortical and subcortical hierarchies, down to the intracellular mechanisms that underwrite synaptic processes-and possibly subcellular predictive coding mechanisms: e.g., (Kiebel and Friston, 2011, Furutachi et al., 2024, Shaikh and Assisi, 2025).

The above implies the following taxonomy of principles and processes we have encountered so far. The free energy principle is a means of specifying the behavior of systems in terms of the ‘beliefs’ one part of that system holds about another. This formulation turns upon an internal model, against which free energy is evaluated. The notion of internal generative models-to understand perception and behavior-in a broad, philosophical, setting underwrites the field of predictive processing in which one seeks to align an internal model to an external world. Active inference emerges from the free energy principle, when bringing about this alignment through action. When one commits to specific forms for the generative model, one arrives at theories of brain function. For instance, a commitment to Gaussian fluctuations implies predictive coding. At this point, it is possible to express belief-updating through a series of differential equations (e.g., Bayesian filters), whose solutions furnish the predictions and errors one would expect to see under predictive coding. One can then go further and propose process theories, about the instantiation of predictive coding in neuronal networks.

The equations in Figures 2 and 4 are special cases of the paths of least action in Figure 6. Their functional form equips predictive coding with the ability to make some clear and principled predictions about what one would expect to see empirically, at various scales in the brain. For example, one would expect to see some form of hierarchical anatomy, within which neuronal dynamics couple hierarchical levels in a recurrent fashion (Zeki and Shipp, 1988; Felleman and Van Essen, 1991; Hilgetag et al., 2000). The requisite forward and backward neuronal message-passing should show some fundamental asymmetries in terms of their sources and targets (e.g., laminar specificity in the cortex) (Hilgetag et al., 2000; Shipp, 2005; Buffalo et al., 2011; Sherman and Guillery, 2011; Markov et al., 2013). Dynamically, one would predict that forward connections are linear or *driving* in nature because the dynamics (black box equations in Figure 2) are linear in the prediction errors. Conversely, we would expect the backward predictions to be nonlinear and modulatory (Nealey and Maunsell, 1994; Angelucci and Bressloff, 2006; Zikopoulos and Barbas, 2007), because the dynamics of prediction errors (red box equations in Figure 2) are nonlinear in the predictions.

However, predictive coding goes further than heuristic notions of providing top-down, modulatory or contextual guidance. This is because the nonlinearities in predictive coding come in two separable flavors. The first is due to nonlinearities in the generative model (see Figure 2). In other words, latent states interact and conspire in a nonlinear way to generate sensations. One simple example is visual occlusion (Rao, 1999; Adams et al., 2012). This kind of nonlinearity is distinct from the precision-weighting of the prediction errors that can be associated with attentional selection. This distinction is highlighted in Figure 4 that considers predictions of precision, which themselves predict things like representational sharpening, and related phenomena such as biased competition (Desimone, 1998; Spratling, 2008; Feldman and Friston, 2010; Lee et al., 2013). This architecture suggests that thalamocortical projections instantiate precision or gain control, where: “This selective amplification is implemented by a cooperative mechanism requiring thalamic input from the pulvinar” (Furutachi et al., 2024). Interestingly, a similar parallel or dual architecture – mediating predictions of content and precision, respectively – is found in thalamocortical projections to the prefrontal cortex: “The largely parallel thalamo-cortical pathways terminated among distinct and laminar-specific neurochemical classes of inhibitory neurons that differ markedly in inhibitory control” (Zikopoulos and Barbas, 2007). In turn, this implicates inhibitory interneurons in the deployment of precision. See Barron et al. (2020) for a related discussion of descending pathways from hippocampus.

One can now go further and make predictions about the physiological concomitants of these functional asymmetries. For example, if forward connections are largely linear (i.e., driving) and backward connections are nonlinear (i.e., modulatory), one would expect a period halving or increase in the characteristic frequencies of forward, relative to backward afferents (Friston K., 2008). This prediction was subsequently verified empirically and is now almost a predictive coding meme: e.g., (Mejias et al., 2016; Michalareas et al., 2016). Predictions that have subsequently been confirmed are not restricted to neurophysiology. For example, in neuroanatomy: “a neural inference arising from the earliest formulations of predictive coding is that the source populations of forward and backward pathways should be completely separate, given their functional distinction; this aspect of circuitry – that neurons with extrinsically bifurcating axons do not project in both directions – has only recently been confirmed.” (Shipp, 2016) p. 1792. As time has passed, the predictions furnished by predictive coding have progressively verified with careful psychophysical and electrophysiological studies of increasing resolution and mechanistic detail: e.g., (Furutachi et al., 2024). For example, predictions that neurons encode prediction errors are now being verified empirically at the single unit level: e.g., (Liu and Buonomano, 2025).

## 9 Predictive processing everywhere: planning and the consequences of action

Predictive processing – read as active inference – comes in two flavors. First, there is *mere active inference* or active predictive coding, in which the generative model simply supplies prior predictions that are fulfilled by reflexes (Friston et al., 2010a; Friston, 2011; Adams et al., 2013a; Rao et al., 2023): See Figure 6 for an example. The other kind – used by you and me – turns on generative models *that include the consequences of action*. This equips certain natural kinds with the ability to plan and select among courses of action (that minimize *expected free energy*) (Parr et al., 2022; Friston et al., 2023). In the technical literature, this distinction is read as a difference between *control* (Powers, 1973; Kappen et al., 2012) and *planning* as inference (Attias, 2003; Botvinick and Toussaint, 2012; Lanillos et al., 2021).

A key distinction is that planning toward intended goals entails the selection among a number of discrete policies or paths into the future (where the expected free energy scores the probability of selecting a particular policy – see below). In this setting, prediction takes on its vernacular meaning as predicting the future (as opposed to predicting what I would see, if I knew the causes of my sensations). A subtle aspect of the generative models required for planning as inference – and ensuing action selection – is that one has to select from a number of discrete policies. This brings us to the final generalization of predictive processing; namely, from *continuous* to *discrete* representations.

The foundational role generative models entailed by predictive processing and inference cannot be overstated. But what kind of generative model? Previously, we have limited the discussion to continuous state-space models. But are these the most apt for describing the brain? One could argue that – at least in some parts of the brain – discrete state-space models are more appropriate. Discrete models basically carve nature at its joints by assuming that the state of the world can be in one of a finite number of states. In factorial generalizations, there can be multiple factors with that can interact to generate outcomes (or states at a lower hierarchical level). In computational neuroscience, these are generally referred to as partially observed Markov decision processes (POMDP) and have been the workhorse of many advances in active inference (Parr and Friston, 2018a,c; Da Costa et al., 2020; Parr et al., 2022), when applied to choice behavior and belief updating. So, what is the evidence for discrete representations in the brain?

Perhaps the clearest example is the existence of receptive fields. For example, place cells (Moser et al., 2008) tell one immediately that the encoding of location in the brain is not in terms of continuous (e.g., GPS) coordinates but in terms of discrete representations; namely, “I am here”. One can generalize this notion to any receptive field. For example, motion-sensitive neurons in the visual hierarchy do not respond more vigorously to faster optical flow. They have a preferred velocity, effectively representing hidden states over a number of mutually exclusive generalized motions. If one generalizes the notion of predictive coding to cover discrete state-space models, one can now interpret neuronal dynamics (and plasticity) in terms of schemes called *belief propagation* and *variational message passing* (Kschischang et al., 2001; Lee and Mumford, 2003; Winn and Bishop, 2005; Yedidia et al., 2005; Friston et al., 2017a,c).

The functional form of the ensuing inference and learning schemes is isomorphic to predictive coding but with some subtle differences (Friston et al., 2017c) that could have implications for how we test mechanistic hypotheses about neuronal implementation. One interesting example is that canonical microcircuit formulations of predictive coding (Mumford, 1992; Rao and Ballard, 1999; Bastos et al., 2012) assume distinct neurons encode precision-weighted prediction errors and that these drive representational units encoding expected latent states: see Figure 1. However, in discrete schemes, a more biologically plausible hypothesis is that prediction errors are encoded by postsynaptic currents, while expectations are encoded in terms of depolarization and subsequent activity or firing (Friston et al., 2017a). This provides a nice example of hypotheses that could be disambiguated empirically; namely, are prediction errors encoded by the activity of distinct neurons or by the distinct activities of any neuron? In summary, “one can think about process theories for variational treatments of Markov decision processes as formally similar to equivalent process theories for state-space models, such as predictive coding (Rao and Ballard, 1999; Bastos et al., 2012). There are some finer, neurobiologically plausible details of the dynamics of expectations about hidden states that we will consider elsewhere. For example, activity-dependent (e.g., NMDA-R dependent) depolarization that underwrites excitation-inhibition balance” (Friston et al., 2017a).

Another interesting question is the encoding of precision in discrete state-space models. At present, there are two ways of encoding probabilistic mappings in terms of synaptic connection strengths. The first rests upon something called a Dirichlet encoding in which there are, effectively, tags that are accumulated in an experience-dependent way and then re-normalized to produce likelihood mappings and transition probabilities (Friston et al., 2016). Again, this is a biologically plausible scheme in relation to associative plasticity and accompanying decay terms. However, it does not accommodate context sensitive or fast fluctuations in precision of the sort associated with attention (Parr and Friston, 2018b; Friston, 2019). This kind of precision control may involve a different (Gibbs) kind of parameterisation that has been associated with classical neuromodulators (Parr and Friston, 2017).

Functionally, the benefit of discrete state generative models is that they greatly simplify planning and decision making – which both depend upon selection between alternative courses of action one could pursue. This raises an interesting question. If one’s generative model includes several plausible future trajectories and their consequences, how does one draw inferences about these future trajectories for which no data are yet available? In the absence of informative data, beliefs about what to do next must be determined by prior beliefs. This is where expected free energy comes in (Friston et al., 2015). Expected free energy is a functional (function of a function) of our current beliefs that acts as a potential that scores the implausibility of alternative futures. In effect, it determines the sort of creature we believe ourselves to be and the kinds of behaviors we expect ourselves to engage in.

While superficially similar in form to variational free energy (see Figure 7), expected free energy scores how risky and ambiguous a policy is. Risk, here, is how far one expects a trajectory to deviate from (prior) preferred outcomes, while ambiguity scores the uncertainty with which sensory outcomes are predictable under the hidden states predicted along that path into the future. Another way of putting this is that we expect to behave to achieve preferred outcomes whilst also selecting those data that help resolve our uncertainty about hidden states (Mirza et al., 2016; Heins et al., 2020), parameters (Friston et al., 2017b; Kaplan and Friston, 2018), and structures (Friston et al., 2025) in our generative model. It is to the last of these that we turn in our concluding section.

**FIGURE 7:**
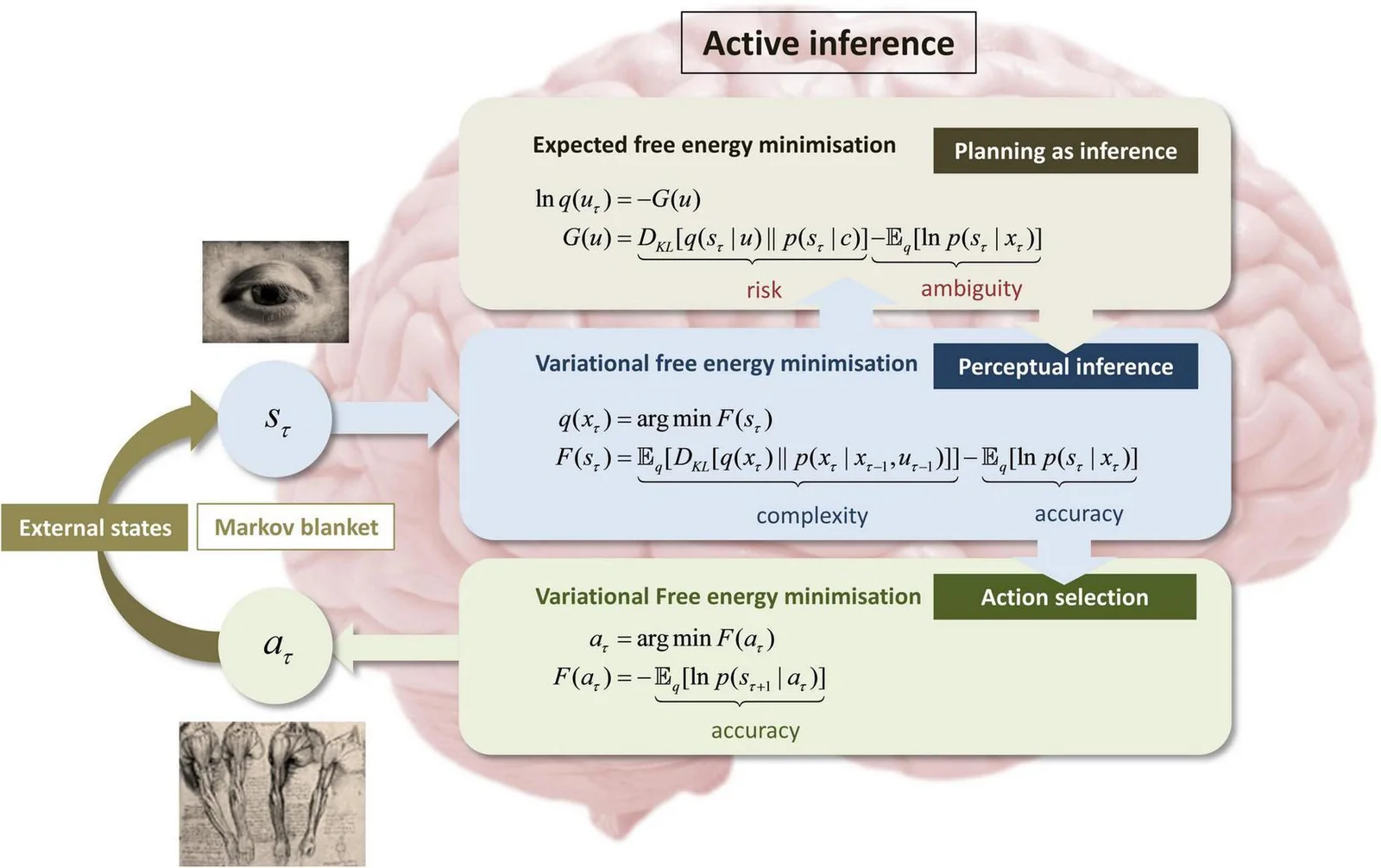
Expected free energy and active inference: This graphic, adapted from (Friston et al., 2022) under a CC BY-NC 4.0 license, illustrates the relationship between variational and expected free energies in determining behavior. Minimization of variational free energy, through predictive coding or its discrete state-space analog, supplies a set of current beliefs under which expected free energy for alternative future trajectories can be determined. Such trajectories are assigned prior plausibility based upon their negative expected free energy. These priors allow one to draw inferences about which path to follow. The result is behavior that has both explorative and exploitative elements, resolving uncertainty about the world while seeking those outcomes that one assigns a high prior probability (i.e., preference). This speaks to a circularity in the definition of preferences and rewards. While in reinforcement learning, one thinks of a behavior as motivated by a reward, it is equally valid to define a reward as the outcome(s) one would achieve under the most plausible behavior. Active inference makes this explicit through *a prior* distribution over future outcomes that contributes to the ‘risk’ in expected free energy. In this scheme, posterior beliefs about control states (*u*) provide empirical priors or inductive biases on Bayesian beliefs about states of the world (*x*) – and their sensory consequences (*s*), which are fulfilled by actions (a) that maximize the accuracy part of variational free energy (e.g., minimize proprioception prediction errors).

## 10 Structured predictions: causal discovery in the brain

There are clearly many avenues one could pursue under the predictive processing framework. We close with one important issue – structure learning – that is engaging many people in neuroscience (Kemp and Tenenbaum, 2008; Gershman and Niv, 2010; Tenenbaum et al., 2011; Tervo et al., 2016; Rutar et al., 2022) and beyond (Friedman and Koller, 2003; Pellet and Elisseeff, 2008; George et al., 2021; Rutar et al., 2022; Da Costa et al., 2025). Intuitively, structure learning can be understood as the process by which one discovers the best internal model to use. Unlike the forms of predictive processing discussed above, this means one cannot assume a set number of variables that generate predictions, or even that variables are related, such that one can be used to make predictions about the other. So how does structure learning relate to predictive processing? Under the above arguments, predictive processing applies at multiple scales and to kinds of variables. When viewed through the lens of minimizing variational free energy, a key issue comes into focus: variational (a.k.a., approximate) Bayesian inference is defined in terms of a *mean field approximation*, in which different kinds of unknowns are represented as marginal posteriors. This may sound complicated but is the *raison d’etre* for using variational calculus in active inference; i.e., one unknown contextualizes belief updating about another. For example, one has to learn the right parameters or synaptic connections in order to optimize state-estimation or inference. Conversely, one has to have the right inference in order to supply prediction errors that are accumulated during learning. See Figure 2.

The mean field approximation rests on a partition of the unknown attributes of a generative model into *states* that change quickly, *precision* that contextualizes inference, and slowly changing *parameters*. However, there is a further attribute that we have not addressed; namely, the *structure* of the model; i.e., architecture and functional form of its parameterization. The distinction between model *states*, *parameters* and *structure* leads to a distinction between *inference*, *learning* and *selection*. But why selection? In variational inference, each structure corresponds to a particular model or hypothesis that could best explain (sensory) data. To disambiguate among different models or hypothesis, one simply selects the model with the greatest evidence (or smallest variational free energy). This is known as Bayesian model selection. Indeed, one could argue that this is the mathematical image of natural *selection* at an evolutionary timescale (Frank, 2012; Campbell, 2016; Vanchurin et al., 2022) or attentional *selection* during perceptual inference (Bosman et al., 2012; Parr et al., 2020) or, indeed action *selection* during active inference (Cisek, 2007; Frank et al., 2007; Rao, 2010).

Another view of Bayesian model selection is afforded by causal discovery and structure learning (Kemp and Tenenbaum, 2008; Gershman and Niv, 2010; Tenenbaum et al., 2011; Tervo et al., 2016; Rutar et al., 2022). The structure learning or model selection problem is a challenging problem because it calls upon the exploration of a model or hypothesis space that is not straightforward to specify or explore. There are generally two approaches to the model selection problem; namely, a top-down approach in which one starts with an overly expressive model and prunes away redundant parts, through things like Bayesian model reduction and synaptic pruning (Tononi and Cirelli, 2006; Srivastava et al., 2014; Da Costa et al., 2025). Alternatively, one can grow a model *de novo*, under the principles of least action and redundancy. This brings us back to where we started in terms of compression and maximizing mutual information under constraints. In some senses, many current endeavors in systems neuroscience – e.g., (Whittington et al., 2019; Bakermans and Behrens, 2025) – could be viewed as discovering the right structures that constitute our generative models; namely, carving nature at its joints in the right way. Although this takes us a long way from the original conception of predictive coding, it foregrounds the foundational role of hypotheses or generative models; both in terms of neuroscientific enquiry and the subject of that enquiry; namely, ourselves.

## 11 Conclusion

Bring this right back to the beginning, each of the themes we have encountered here draw from the phantastic properties of our brains. Predictive coding and its generalizations formalize the notion that perception (and action) are constructive endeavors that make sense of reality through finding the explanations from which that reality can best be generated. Internal generative models thereby offer a unifying synthesis of the developments from Helmholtz’s unconscious inference, through 20th century developments in variational calculus, to the mechanics of predictive coding. They are just as central to modern extensions into predictive processing and active inference, in addition to underwriting generative artificial intelligence and machine learning. From the perspective of theoretical neurobiology, predictive coding has taught us that to understand the brain, the fundamental problem one must address is how the brain understands the world.
